# Symptoms and management of cow's milk allergy: perception and evidence

**DOI:** 10.3389/falgy.2024.1348769

**Published:** 2024-06-13

**Authors:** E. Robert, H. A. Al-Hashmi, A. Al-Mehaidib, K. Alsarraf, M. Al-Turaiki, W. Aldekhail, W. Al-Herz, A. Alkhabaz, Khalid O. Bawakid, A. Elghoudi, M. El Hodhod, Ali A. Hussain, Naglaa M. Kamal, L. T. Goronfolah, B. Nasrallah, K. Sengupta, I. Broekaert, M. Domellöf, F. Indrio, A. Lapillonne, C. Pienar, C. Ribes-Koninckx, R. Shamir, H. Szajewska, N. Thapar, R. A. Thomassen, E. Verduci, C. E. West, Y. Vandenplas

**Affiliations:** ^1^ UZ Brussel, KidZ Health Castle, Vrije Universiteit Brussel (VUB), Brussels, Belgium; ^2^ Pediatric Department, King Abdulaziz Hospital, Jeddah, Saudi Arabia; ^3^ Department of Pediatrics, King Faisal Specialist Hospital & Research Center, Riyadh, Saudi Arabia; ^4^ Department of Pediatric Gastroenterology and Hepatology, Al Amiri Hospital (MOH), Kuwait City, Kuwait; ^5^ Department of Pediatric Gastroenterology and Hepatology, Dar Al Shefaa Hospital (PVT), Hawally, Kuwait; ^6^ Department of Pediatrics, King Salman Hospital, Riyadh, Saudi Arabia; ^7^ Section of Gastroenterology and Hepatology, Department of Pediatrics, King Faisal Specialist Hospital and 12 Research Centre, Riyadh, Saudi Arabia; ^8^ Department of Pediatrics, College of Medicine, Kuwait University, Kuwait City, Kuwait; ^9^ Department of Pediatrics, Allergist & Clinical Immunology, Mubarak AlKabeer Hospital, Jabriya, Kuwait; ^10^ Department of Pediatrics, MCH Hospital Jeddah, Jeddah, Saudi Arabia; ^11^ Sheikh Khalifa Medical City, Abu Dhabi, United Arab Emirates; ^12^ CMHS, UAE University, Abu Dhabi, United Arab Emirates; ^13^ Faculty of Medicine, Ain Shams University, Cairo, Egypt; ^14^ Department of Pediatrics, Al Adan and Al Salam International Hospitals, Kuwait City, Kuwait; ^15^ Department of Pediatrics & Pediatric Hepatology, Kasr Alainy Faculty of Medicine, Cairo University, Cairo, Egypt; ^16^ College of Medicine, King Saud Bin Abdulaziz University for Health Sciences, Jeddah, Saudi Arabia; ^17^ Department of Pediatrics, American Hospital Dubai, Dubai, United Arab Emirates; ^18^ Department of Pediatrics, NMC Specialty Hospital, Dubai, United Arab Emirates; ^19^ Department of Pediatrics, Faculty of Medicine and University Hospital Cologne, University of Cologne, Cologne, Germany; ^20^ Department of Clinical Sciences, Pediatrics, Umeå University, Umeå, Sweden; ^21^ Department of Pediatric University of Salento, Lecce, Italy; ^22^ Neonatal Intensive Care Unit, Necker-EnfantsMalades Hospital, Paris University, Paris, France; ^23^ CNRC, Department of Pediatrics, Baylor College of Medicine, Houston, TX, United States; ^24^ Department of Pediatrics, “Victor Babes” University of Medicine and Pharmacy, Timisoara, Romania; ^25^ Coeliac Disease and Gastrointestinal Immunopathology Research Unit, Hospital La Fe Research Institute Valencia, Valencia, Spain; ^26^ Institute of Gastroenterology, Nutrition and Liver Diseases, Schneider Children’s Medical Center, Faculty of Medicine, Tel-Aviv University, Tel Aviv, Israel; ^27^ Department of Pediatrics, The Medical University of Warsaw, Warsaw, Poland; ^28^ Department of Gastroenterology, Hepatology and Liver Transplant, Queensland Children's Hospital, Brisbane, QLD, Australia; ^29^ School of Medicine, University of Queensland, Brisbane, QLD, Australia; ^30^ Woolworths Centre for Child Nutrition Research, Queensland University of Technology, Brisbane, QLD, Australia; ^31^ Department of Pediatrics, UCL Great Ormond Street Institute of Child Health, London, United Kingdom; ^32^ Division of Pediatric and Adolescent Medicine, Department of Pediatric Medicine, Oslo University Hospital, Oslo, Norway; ^33^ Department of Pediatrics, Vittore Buzzi Children's Hospital University of Milan, Milan, Italy

**Keywords:** cow's milk allergy, extensively hydrolysed formula, amino acid formula, soy formula, hydrolysed rice formula, probiotic, prebiotic, synbiotic

## Abstract

**Introduction:**

The diagnosis and management of cow's milk allergy (CMA) is a topic of debate and controversy. Our aim was to compare the opinions of expert groups from the Middle East (*n* = 14) and the European Society of Paediatric Gastroenterology, Hepatology and Nutrition (ESPGHAN) (*n* = 13).

**Methods:**

These Expert groups voted on statements that were developed by the ESPGHAN group and published in a recent position paper. The voting outcome was compared.

**Results:**

Overall, there was consensus amongst both groups of experts. Experts agreed that symptoms of crying, irritability and colic, as single manifestation, are not suggestive of CMA. They agreed that amino-acid based formula (AAF) should be reserved for severe cases (e.g., malnutrition and anaphylaxis) and that there is insufficient evidence to recommend a step-down approach. There was no unanimous consensus on the statement that a cow's milk based extensively hydrolysed formula (eHF) should be the first choice as a diagnostic elimination diet in mild/moderate cases. Although the statements regarding the role for hydrolysed rice formula as a diagnostic and therapeutic elimination diet were accepted, 3/27 disagreed. The votes regarding soy formula highlight the differences in opinion in the role of soy protein in CMA dietary treatment. Generally, soy-based formula is seldom available in the Middle-East region. All ESPGHAN experts agreed that there is insufficient evidence that the addition of probiotics, prebiotics and synbiotics increase the efficacy of elimination diets regarding CMA symptoms (despite other benefits such as decrease of infections and antibiotic intake), whereas 3/14 of the Middle East group thought there was sufficient evidence.

**Discussion:**

Differences in voting are related to geographical, cultural and other conditions, such as cost and availability. This emphasizes the need to develop region-specific guidelines considering social and cultural conditions, and to perform further research in this area.

## 1 Introduction

Cow's milk allergy (CMA) is one of the most prevalent food allergies in infants and children under the age of 3 years (1–4). CMA is an immune-mediated hypersensitivity response to several proteins in cow's milk (CM), mainly casein and lactoglobulin. The reported prevalence in Europe ranges from <1% to 5%, while a prevalence of 3.4% is reported in the Middle East (5–9). CMA can be IgE-mediated, non-IgE-mediated, or mixed. Depending on the type of immunological responses, the clinical manifestations are classified as immediate or delayed (9). Making an accurate diagnosis followed by appropriate treatment is crucial to prevent over- and under-diagnoses and consequently over- and under-treatment. This is a real challenge due to the lack of specific symptoms or an accurate diagnostic test (7, 8). Early diagnosis is a key factor, as delaying the diagnosis of CMA can lead to faltering growth and malnutrition (9).

An expert group of the European Society of Paediatric Gastroenterology, Hepatology and Nutrition (ESPGHAN) has recently published consensus recommendations regarding the diagnosis and management of cow's milk allergy in children (10). Since food allergy and its management are significantly influenced by social contexts, eating habits, and available resources, the purpose of this article is to compare the opinions of a group of Middle East expert with those of ESPGHAN and thereby examine regional differences (11).

## 2 Materials and methods

An ESPGHAN expert group (*n* = 13) has developed a consensus paper on the diagnosis and management of CMA (10, 12). This paper summarizes the most important findings and recommendations from systematic reviews and meta-analyses regarding the prevalence, pathophysiology, symptoms and diagnosis of CMA. The statements circulated three times before consensus was reached that the statements could be voted on. There was one voting round, which was on-line and anonymous.

The authors of the ESPGHAN voted on these statements (10), whereas a Middle-Eastern expert group (*n* = 14) voted on a selection of statements. Each statement was given a score from 0 to 9. A score of 6 or higher indicated agreement, while a score of five and less indicated disagreement. The higher the score, the greater the degree of agreement.

The Middle East meeting was funded by Abbott Nutrition MENAP, who invited the participants, and covered meeting and publication costs. The Middle East participants (all paediatric gastroenterologists, except one allergologist) were asked to vote only on the statements that were selected by the first author of the ESPGHAN position paper (YV). Limitation of time necessitated a selection of the statements. The Middle East group voted anonymously during a face-to-face meeting. The votes of both expert groups were collected in a common file, analysed descriptively and the median and mean were calculated. In addition to the range, the highest and lowest scores were provided, as well as the number of disagreements scoring five or less.

## 3 Results

The majority of experts agreed that symptoms of crying, irritability and colic as isolated manifestations are not suggestive of CMA. Although the four statements regarding this topic were accepted by both groups, the Middle Eastern group was more in favour for colic as a possible symptom of CMA. The ESPGHAN authors strongly supported that colic, by itself, is not a symptom of CMA, and, therefore, were less supportive of a time limited elimination diet for infantile colic than the Middle Eastern group. In the latter there was 1 author with a disagreement for two statements. The results are presented in Table 1.

**Table 1:** Crying and infant colic.

	Mean/median Range (Number of disagreements)	
	M East (*n* = 14)	ESPGHAN (*n* = 13)
In infants who present with crying and irritability, there is insufficient data to recommend a time-limited CM elimination diet followed by an OFC	7.6/8 5–9 (1)	8.4/9 6–9
There is insufficient data to support infant colic occurring as a single manifestation of CMA	6.7/8 1–9 (1)	8.4/9 6–9
When treatment for infant colic, fulfilling Rome IV clinical research criteria, is considered, and where CMA is suspected based on additional symptoms, a time limited elimination diet can be trialled which should be followed by an OFC	8.4/9 7–9	7.4/9 4–9 (1)

The Middle East group voted on the following statement: “in patients not responding to conventional therapies for functional GI disorders (FGIDs), CMA can be considered and patients trialled on a time limited elimination diet which should be followed by an OFC.” [voting: mean 8.6: median 9 (range 7–9)]. Later the ESPGHAN group changed the wording of this statement to “In patients not responding to other standard treatments for functional abdominal pain disorders, there is insufficient evidence to recommend a time limited CM elimination diet followed by an OFC” [voting: mean 7.9; median 8 (range 6–9)]. Because of the different wording, this statement was not cited in Table 1.

Regarding the diagnostic elimination diet, the Middle Eastern group reached a higher consensus than the ESPGHAN group, as seen in Table 2. Both groups recommend to restrict the use of amino acid formula (AAF) for severe cases, including patients with severe malnutrition and, therefore, do not recommend a step-down approach, starting with AAF as diagnostic elimination diet. Three out of the 27 experts from the combined groups, however, did not agree to recommend a CM based extensively hydrolysed formula (eHF) as first choice for a diagnostic elimination diet. Similarly, three out of the 27 experts (2 from ESPGHAN and 1 from Middle East) disagreed to recommend rice hydrolysed formula (RHF) as an alternative first choice option for a diagnostic elimination diet. The rate of disagreement was similar for soy infant formula. Obviously, unanimous agreement could not be reached regarding the different options as “first choice” diagnostic elimination diet since the same frequency of disagreement was found for each option. It can, therefore, be concluded that more data are needed to provide clear recommendations regarding the most adequate diagnostic elimination diet.

**Table 2:** Diagnostic and therapeutic elimination diet.

	Mean/median Range (Number of disagreements)	
	M East (*n* = 14)	ESPGHAN (*n* = 13)
In formula fed infants, a CM based extensively hydrolysed formula (eHF) is the first choice for a diagnostic elimination diet in mild/moderate cases	8.1/8.5 4–9 (1)	7.2/9 0–9 (2)
In formula fed infants, amino acid-based formula (AAF) for a diagnostic elimination diet should be reserved for severe cases or patients with severe malnutrition	8.3/8.5 6–9	8.5/9 7–9
Although some consensus papers recommend a step-down approach using AAF as diagnostic elimination diet in every infant suspected of CMA, there is insufficient evidence for this recommendation	8.0/9 6–9	8.6/9 6–9
Although less studied than CM based eHFs, rice hydrolysed formulae (RHFs) can be considered as an alternative for a diagnostic elimination diet	8.1/9 5–9 (1)	7.4/8 1–9 (2)
Soy infant formula should not be used as the first choice for diagnostic elimination diet but can be considered in some cases for economic, cultural and palatability reasons	8.0/9 5–9 (1)	7.6/9 0–9 (2)
Rice hydrolysed formula can be considered as an alternative to CM based eHF for a therapeutic elimination diet	8.2/9 6–9	7.8/8 5–9 (2)

The statement regarding the absence of an added therapeutic efficacy of probiotic, prebiotics and synbiotics to eHFs and AAFs was accepted by all European authors but was rejected by 3/14 Middle Eastern authors (Table 3).

**Table 3:** Pro-, pre- and synbiotics.

	Mean/median Range (Number of disagreements)	
	M East (*n* = 14)	ESPGHAN (*n* = 13)
There is insufficient evidence demonstrating that the addition of probiotics, prebiotics or synbiotics to eHFs and AAFs improves their therapeutic efficacy	6.6/8 1–9 (3)	8.9/9 9

## 4 Discussion

Overall, all statements were accepted by both groups of experts. However, some key differences were observed. Several organisations, such as The British Society for Allergy & Clinical Immunology (BSACI), The World Allergy Organisation and ESPGHAN encourage the development of region-specific guidelines that meet the needs of children from all social strata in the targeted countries (11–14).

CMA symptoms and indicators include cutaneous, gastrointestinal, respiratory, and systemic responses. IgE mediated CMA's clinical symptoms appear “immediately” and occur within minutes to 2 h. In a non-IgE-mediated immune reaction, the appearance of symptoms is “delayed”, and develop usually after ≥2 h up to 1 week after exposure (10, 15). The majority of experts in both groups agreed that symptoms of crying, irritability and colic as single manifestations are not suggestive of CMA. However, the Middle Eastern experts tended to consider CMA as a cause of infant distress and colic compared to the European authors.

The diagnosis of CMA is based on a thorough history and physical examination. A diagnostic elimination of CM protein for 1–4 weeks followed by an oral food challenge or reintroduction of CM is advised when CMA is suspected (10).

In non-exclusively breastfed infants suspected to suffer from CMA, a formula with reduced allergenicity for CM is recommended. However, formula selection is controversial and frequently influenced by availability and economic factors, as well as scientific evidence (1, 2, 11, 16). At the current time, evidence is strongest for the use of a CM-based eHF for the diagnostic elimination diet. The combined groups, however, failed to reach unanimous consensus on the use of eHF as the first-choice diagnostic elimination diet”, given 3/27 authors disagreed. One rationale for recommending eHF as the first-line formula is the higher cost of AAF in the majority of countries (17). Nonetheless, some studies have found AAF to be comparable or more cost-effective (11), and recommend a step- down approach. Consequently, there is a need for international guidelines to be adapted to national recommendations that take into consideration the local health system (16).

The statement that AAF should be reserved for severe cases or patients with serious malnutrition was accepted by both expert groups. A recent review of Ribes-Koninckx et al. confirms this statement and suggests the use of AAF when treatment with eHF is unsuccessful or in the case of severe CMA, particularly with associated nutritional deficiencies (3). IgE-mediated anaphylaxis associated with CMA, acute and chronic food protein induced enterocolitis syndrome (FPIES), multiple food allergies associated with CMA, and eosinophilic esophagitis unresponsive to a prolonged exclusion diet are a few examples (3). Five to ten percent of children with an IgE-mediated CMA react to eHF (2, 3). AAF, compared to eHF, does not contain immunogenic peptides that stimulate the immune system. Due to these negative factors, CMA and the use of AAF must be frequently re-evaluated. Patient's age at diagnosis, symptoms, serum IgE level, and nutritional status are involved in this reassessment (3).

Some guidelines propose a step-down strategy. In this case, AAF is used as diagnostic elimination diet. Subsequently, an eHF is used as the therapeutic elimination diet when the OFC or reintroduction caused a relapse of symptoms. However, this approach is not frequently used, mainly for economic reasons (10).

In Europe, formulas containing hydrolysed rice protein (HRF) have been commercially available since the 2000s as a nutritionally acceptable and well-tolerated plant-based alternative to eHF (2). Access to and availability of HRF are the primary factors limiting its global usage; eHFs and AAFs are the two most accessible types of substitute formulas (11). In addition to regional differences, there are differences in usage between specialists and non-specialists (2). This difference could not be demonstrated in the current manuscript because opinions of non-specialist were not collected.

One advantage of HRF is that it has a better flavour than eHF. In addition, it does not contain any residues of CM protein (1). In contrast, concerns have been raised regarding the arsenic content of infant rice products (18). Arsenic can be found in small amounts in rocks, soil, and groundwater, both in its inorganic form and in organic forms (19). Consequently, exposure during infancy may have potential long-term health effects like an increased risk for developing pulmonary disease and cancer in adulthood (19, 20). Since 2016, the European Union has set a maximum concentration of 0.10 mg/kg for inorganic arsenic in rice intended for infants under the age of 3 (21). Hojsak et al. concluded that the arsenic concentration in HRF is low and well within the safe range established by the European Food Safety Authority, with no significant difference compared to the arsenic concentration in cow's milk formulas (19, 22). However, it should be noted that not all commercially available HRF list the arsenic content on their labels (23). The arsenic con- centration in water used to prepare the formulae, will also contribute to final arsenic content. The statements regarding a role for HRF as a diagnostic elimination diet were accepted by both expert groups despite the paucity of data.

Soy formula may also be a treatment option but there is limited evidence to support its usage (10). Current soy infant formulae are nutritionally adequate and promote healthy growth and development. In the first 2 years of life, soy infant formula does not reduce the likelihood of allergic manifestations (24, 25). Cross-allergy between CM protein and soy is rare in IgE-mediated CMA, and, therefore, soy-based infant formula can be used as an alternative therapy diet (26, 27). However, it should be noted that cross-allergy is more prevalent in non-IgE-mediated CMA. The majority of data supporting this association originate from the United States. Studies conducted in the US indicate that 30%–50% of children with FPIES react to both cow's milk and soy, while the overwhelming majority of studies originating from outside the US indicate a much lower percentage (10, 28). Soy allergy prevalence ranges from 0% to 0.5% in the general population and 0 to 12.9% in allergic children (29). The availability of soy infant formula in many European countries has decreased in recent years. Soy infant formula can be considered as a second option when other formulae are not possible due to economic or cultural factors. Additionally, soy infant formula has a more favourable flavour and is cheaper compared to eHF (10).

CMA is associated with intestinal dysbiosis, with reduced diversity of gut microbiota as well as a low abundance of Bifidobacteria, Lactobacilli and Bacteroides (30). Modulating the intestinal microbiome may be a valuable management strategy for CMA. Adding pre, pro-, syn-, or postbiotics to infant formulae could be one way to reach this goal (31). Several hypoallergenic formulations include pre-, pro-, syn- or postbiotics. However, due to a lack of evidence-based literature, their additional efficacy in CMA has not yet been established (3, 31). All ESPGHAN experts agreed that there is insufficient evidence to conclude that prebiotics, probiotics and synbiotics increase the efficacy of elimination diets regarding CMA (although there are other benefits suggested such as decreased incidence of infection, decreased episodes of fever, decreased prescriptions of antibiotics..), whereas 3 of the 14 authors of the Middle Eastern group thought there was evidence.

The 2021 Middle East Consensus Statement described that adding prebiotics and synbiotics to a therapeutic formula may improve the tolerance to CM protein by the end of the first year of life (9). Synbiotics can improve gut microbiota in non-IgE-mediated CMA, bringing it closer to that of healthy newborn (9). Sorensen et al. described in their systematic review that the combination of a synbiotic and AAF resulted in the same reduction of allergic symptoms and normal growth as AAF alone (31). In addition, outcomes suggest that the observed combination of improved dysbiosis and a trend towards decreased infection, hospital admissions and antibiotic use might be due to the essential role that the intestinal microbiome plays in maintaining health and disease development (31, 32). Several studies have demonstrated that antibiotic use in children aged 0–3 years causes a less diverse microbiome, with reduced abundance of Bifidobacteria, Lactobcclli and Bacteroides (30, 33). Because of these negative effects on the gut microbiome, antibiotic exposure during the first years of life is associated with an increased risk of developing hay fever, eczema, and food allergy later in life, according to a 2018 meta-analysis (34). Metsala et al. concluded in their study that antibiotic use in children was associated with an increased risk of developing CMA (35). A study encompassing 30,060 children up to 7 years of age, revealed that children who received three or more antibiotic treatments were more likely to develop milk allergy, non-milk food allergy and other allergies compared to children who did not receive antibiotics. The strongest associations were observed at lower ages and varied by antibiotic class (36). Overall, using probiotics during and after an antibiotic course may reduce the harmful impact of antibiotics on the gut flora (34). This could ultimately result in an economic advantage with lower costs (31).

Several additional studies showed that the addition of Lacticaseibacillus rhamnosus GG (LGG) is a cost-effective CMA treatment. In the United States, Guest et al. found that adding LGG to eHCF was a more cost-effective strategy than eHCF alone or AAF, as it improved outcomes at a lower cost (37). A Spanish investigation revealed comparable results (38). A study by Martin et al. indicates that eHCF combined with LGG is the most costeffective strategy for treating CMA in the United Kingdom (39). According to a French study conducted by Paquete et al., the combination of eHCF and LGG was associated with longer symptom-free periods, greater immune tolerance, and reduced costs (40).

Two important limitations of this report are: (i) only the opinion of selected experts was solicited, and (ii) the very restricted selection of statements for the voting of the Middle East group. Gathering input from a broader range of professionals such as general paediatricians, family doctors, allergologists, dietitians, and parents could provide a more comprehensive perspective on how CMA is addressed in clinical practice. Expanding this analysis to a larger population may uncover more noticeable differences between cultures and regions.

## 5 Conclusions

CMA comprises a broad spectrum of symptoms and indicators of varying severity. Because of the limited evidence, some experts differ in opinion regarding the role of crying, irritability and colic in CMA. The optimal diagnostic and therapeutic elimination diets are still debated as well. This emphasizes the importance of developing excellent region-specific guidelines based on the available resources. The approach in CMA should consider the child as an individual, based on clinical contexts. Furthermore, it is essential to consider the social context to ensure that the needs of children from all social strata within the specific countries are met. Moreover, the access to medical services and the availability of CMA-free diet must be taken into account. Emphasis should be made on timely and accurate diagnosis to prevent complications such as growth and developmental issues. Lastly, this study highlights the fact that many current guidelines, including guidelines on CMA, are often based on expert opinion. Knowledge about CMA has increased over the past few decades, however, the study presented here underscores that there is still much work to be done. Through identifying and highlighting discrepancies among expert groups, we have pinpointed research priorities in this area, such as the potential added value of probiotics, prebiotics, and synbiotics for the efficacy of elimination diets. By making substantial progress in these areas over the next few years, we believe we can promote the health and well-being of children suspected of having CMA in the future.

## Data Availability

The original contributions presented in the study are included in the article/Supplementary Material, further inquiries can be directed to the corresponding authors.
